# Creep–Fatigue Behavior and Life Prediction of FGH95 PM Nickel-Based Superalloy Under Different Control Modes

**DOI:** 10.3390/ma19194104

**Published:** 2026-09-25

**Authors:** Yang Liu, Huanhuan Chen, Keke Li, Mengsen Qin, Zhihui Lu, Chuanyong Chen, Haijun Xuan

**Affiliations:** 1 Polytechnic Institute, Zhejiang University, Hangzhou 310015, China; ly_1632026@163.com; 2 AECC Hunan Aviation Powerplant Research Institute, Zhuzhou 412002, China; chenhuan_npu@163.com (H.C.); kekel2025@163.com (K.L.); 3 College of Energy Engineering, Zhejiang University, Hangzhou 310027, China; luzhihui@zju.edu.cn (Z.L.); marine@zju.edu.cn (H.X.); 4 Research Institute of Aero-Engine, Beihang University, Beijing 100191, China; 5 Ocean College, Zhejiang University, Zhoushan 316021, China; qms@zju.edu.cn

**Keywords:** creep–fatigue behavior, FGH95 alloy, strain-controlled mode, stress-controlled mode, life prediction

## Abstract

FGH95 is a powder metallurgy nickel-based superalloy used for aero-engine turbine disks, which can experience combined creep and fatigue loading at elevated temperature. Its response was examined at 620 °C under asymmetric cycling (stress ratio *R* = 0) using maximum strain control from 1.0% to 2.0% and maximum stress control from 1200 to 1430 MPa, each with either 0 or 30 s tensile dwell. Under stress control, the dwell reduced the mean fatigue life by 96.3–98.4%. Under strain control, the response depended on the maximum strain: the measured life changed from 3343 to 3632 cycles at 1.0%, whereas reductions of 36.5–63.5% occurred at 1.2–2.0%. This difference is associated with continuous ratcheting strain accumulation under stress control and progressive stress relaxation under strain control. A unified strain-based life model was calibrated using creep strain rate and inelastic strain range to represent creep–fatigue interaction. For the calibration dataset, all calculated lives fall within a factor-of-two scatter band and show closer agreement than the conventional time fraction rule, whose maximum error factor is 3.69.

## 1. Introduction

Aero-engine turbine disks operate under high-temperature, high-rotational speed, and cyclic loading conditions. Their materials accumulate cyclic fatigue damage, creep damage during load holds, and interaction damage when these processes occur together. The development of safer, more reliable, and longer-lived aero-engines requires more accurate damage assessment and life prediction methods. This need motivates efforts to clarify creep–fatigue coupling in turbine disk materials and to establish more accurate methods for predicting creep–fatigue life.

FGH95 (René 95) is a powder metallurgy (PM) nickel-based superalloy commonly used to manufacture gas turbine disk components for aircraft engines. It can operate at temperatures approaching 700 °C and provides high tensile strength, fatigue resistance, and stress rupture resistance. Previous studies have examined its mechanical properties and influencing factors through experiments, simulations, and mechanism-based analyses. Tao et al. [1] summarized the manufacturing process and microstructural characteristics of FGH95. Tian et al. [2] investigated the deformation characteristics of FGH95 under pure creep using microstructural observations. Their results showed strong sensitivity to applied stress and temperature and indicated that severe deformation may subdivide wide grain boundary regions into finer grains. They subsequently investigated the effects of hot isostatic pressing temperature and heat treatment on the microstructure and creep properties of FGH95 [3,4,5]. These studies reported that dislocation pile-ups near grain boundaries can cause stress concentration and promote microcrack initiation and propagation. They also showed that the precipitation of the γ′ phase and fine carbide particles within grains and along grain boundaries depends on the processing temperature and can enhance the creep resistance of the alloy. Qin et al. [6] investigated the elastoplastic behavior of FGH95 at 620 °C under asymmetric cyclic loading and proposed a modified constitutive model incorporating variable dynamic recovery to predict cyclic stress–strain responses under strain control. Bernstein [7] conducted strain-controlled fatigue tests, including several strain-hold tests and dual-rate continuous-cycling tests, at 650 °C. Four creep–fatigue interaction models were evaluated, but none adequately predicted the fatigue life of René 95 at 650 °C. Li et al. [8] examined the low-cycle-fatigue behavior of FGH95 at 420, 510, and 600 °C and reported temperature-dependent changes in fracture mode and dislocation motion. Hormozi et al. [9] conducted fully reversed strain-controlled creep–fatigue tests on 316 stainless steel at 350 °C. They used a constitutive model based on isotropic and nonlinear kinematic hardening rules to reproduce the cyclic response, and combined it with a time fraction creep damage model for life prediction.

FGH95 PM-superalloy turbine disks operate under the combined effects of high temperature and centrifugal loading and experience both cyclic loading and sustained load holds. Creep–fatigue interaction is therefore an important potential failure mode. In particular, accurate prediction of creep–fatigue damage and life in the tenon-groove region at the disk rim is essential for reliable design and safe operation. The local stress state in this region is not represented by ideal stress or strain control alone, but involves constraints associated with both modes; this makes damage evaluation and life prediction more difficult. Numerous studies have examined creep–fatigue behavior under individual or hybrid control modes. Chen et al. [10] investigated strain-controlled low-cycle fatigue and creep–fatigue interaction in GH4169 at 650 °C. Tensile strain holds reduced fatigue life, with the hold-time effect approaching saturation at 120 s, and cyclic softening depended on both strain amplitude and hold duration. At the crack-propagation scale, Mao et al. [11] studied GH4720Li at 600 and 700 °C and showed that dwell time and temperature altered the dominant fracture mechanism. Their results emphasize the importance of loading history when assessing time-dependent damage in turbine-disk superalloys. Sun et al. [12] conducted symmetric and asymmetric strain-controlled creep–fatigue tests on the nickel-based superalloy GH4169 at 650 °C and observed pronounced tension-compression asymmetry and sustained cyclic softening. Their internal-stress analysis linked cyclic softening and mean stress relaxation mainly to back-stress evolution. Vivek Kalyan et al. [13] studied the creep–fatigue behavior of alloy 617M under different peak-hold modes from 600 to 750 °C. Their comparison showed that stress-hold loading produced a shorter failure life than strain-hold loading. Zhao et al. [14] investigated P92 steel under different dwell times at 630 °C and showed that longer dwell times rapidly reduced fatigue life. For sufficiently long dwells, stress relaxation approached saturation and the residual stress gradually approached the creep-threshold stress. Because stress relaxation can limit the creep-driving stress, conventional strain-controlled creep–fatigue interaction tests may be unable to generate data dominated by creep [15]. Tahir and Liu [16] therefore proposed an experimental loading program to induce creep-dominated creep–fatigue interaction in Inconel 617 at 950 °C. Most of the hysteresis loop was strain controlled, but a tensile-hold segment was added to sustain the creep-driving stress. Zhang et al. [15] further developed this approach as a hybrid stress–strain-controlled creep–fatigue interaction (HCFI) loading method, which permits adjustment of the relative contributions of fatigue and creep damage. Studies of P92 steel using this approach examined creep–fatigue life and the influence of strain amplitude, providing evidence of control path-dependent deformation and damage [17,18]. Zhang et al. [19] studied TP321 austenitic stainless steel under hybrid control; evaluated in detail the effects of strain amplitude, dwell time, hold stress, and temperature; and discussed their influence on creep–fatigue interaction. Kang et al. [20] conducted HCFI tests on Inconel 718 at 650 °C. By changing the hold stress and dwell time, they characterized cyclic response and proposed a method for determining creep-equivalent stress from stress relaxation behavior. Their subsequent overload study showed that the remaining life and deformation response also depended on when the overload was applied [21]. These studies collectively show that the loading control path changes stress relaxation, ratcheting, and time-dependent deformation and therefore must be considered when comparing creep–fatigue life.

Comparing creep–fatigue behavior under stress and strain control is important for understanding the interaction between fatigue and creep. However, because high-temperature creep–fatigue testing is expensive and time-consuming, systematic experimental comparisons covering both modes remain limited. Yuan et al. [22] reported the low-cycle fatigue behavior of 316LN stainless steel under different control modes. Under symmetric strain and stress cycling, the material exhibited amplitude-dependent cyclic hardening or softening and corresponding life characteristics; under asymmetric stress cycling, mean stress and ratcheting strain substantially reduced fatigue life. Li et al. [23] investigated FGH96 at 550 °C using strain-controlled low cycle fatigue tests and stress-controlled tests with and without peak stress holds. They observed amplitude-dependent cyclic softening/hardening and enhanced ratcheting when a tensile hold was introduced under asymmetric stress cycling. These results on another PM nickel-based superalloy reinforce the need to distinguish control mode effects when evaluating the creep–fatigue response of FGH95.

In this study, creep–fatigue tests were conducted on FGH95 for aero-engine turbine disks at 620 °C. The experiments included strain-controlled tests with maximum strains of 1.0–2.0% and stress-controlled tests with maximum stresses of 1200–1430 MPa, using tensile dwell times of 0 and 30 s. Creep–fatigue behavior under the two control modes was evaluated from fatigue life, cyclic response, stress–strain hysteresis loops, stress relaxation, and creep–strain accumulation. A unified strain-based life model was then calibrated for both control modes, and its agreement with the experimental data was compared with that of the conventional time fraction method.

## 2. Materials and Methods

### 2.1. Materials and Specimens

The material investigated in this study was the PM nickel-based superalloy FGH95 provided by Gaona Aero Material Co., Ltd. (Beijing, China), which is commonly used for turbine disks operating under high-temperature and high-rotational-speed conditions. Its nominal chemical composition is listed in Table 1 [6]. The material was solution-treated at 1140 °C for 2 h and then aged at 870 °C for 1 h. All specimens were machined from the rim of a turbine disk blank produced using the same manufacturing process as the service component. Cylindrical specimens with a gauge diameter of 5.5 mm and a gauge length of 14 mm were used for pure creep (PC), pure fatigue (PF), and creep–fatigue interaction (CFI) tests under both strain and stress control, as shown in Figure 1.

### 2.2. Experimental Procedure

Monotonic tensile tests were conducted at 620 °C in accordance with GB/T 228.2-2015, Metallic Materials-Tensile Testing-Part 2: Method of Test at Elevated Temperature [24]. Loading comprised a strain-controlled stage followed by a displacement-controlled stage. During the first stage, strain was measured using a 12 mm extensometer (Instron Corporation, Norwood, MA, USA) at 6 × 10^−5^ s^−1^ to determine the elastic modulus and yield strength. After yielding, the extensometer was removed and the specimen was loaded at a crosshead displacement rate of 7.2 × 10^−4^ mm s^−1^ until complete fracture. Figure 2 shows the tensile curve, and Table 2 summarizes the tensile properties.

All PC, PF, and CFI tests were conducted using an Instron 8801 servo-hydraulic fatigue-testing system (Instron Corporation, Norwood, MA, USA) equipped with a furnace rated to 1000 °C. A test temperature of 620 °C was selected to represent the service condition of the FGH95 turbine disk. Three K-type thermocouples (Instron Corporation, Norwood, MA, USA) were secured with fine steel wire in direct contact with the upper grip surface, lower grip surface, and specimen gauge surface, respectively. The temperature was maintained within ±1 °C during testing. Cui et al. [25] reported that the elastic modulus, tensile yield strength, and plastic modulus of FGH95 show limited sensitivity to strain rate over 10^−4^–10^−2^ s^−1^ between 420 and 650 °C. Accordingly, the strain-loading rate used here, 0.4% s^−1^, lies within the investigated range, whereas the stress-controlled cyclic segments were applied at 0.5 Hz. Fatigue life was defined as the number of cycles to complete specimen fracture.

For the stress-controlled creep–fatigue interaction (SCFI) tests, the triangular loading waveform had a stress ratio of *R* = 0, as illustrated in Figure 3a. Based on the tensile response of FGH95 and the service stresses of the turbine disk, maximum stresses of 1200, 1260, 1340, and 1430 MPa were selected. Dwell times of 0 s, 30 s, and continuous loading were applied at the maximum stress. The 0 s and continuous hold conditions (the latter denoted by ∞) correspond to stress-controlled pure fatigue (SPF) and pure creep tests, respectively. Because stress-controlled fatigue results commonly exhibit appreciable scatter, two replicate tests were conducted for every stress-controlled condition. Table 3 lists the test parameters and all 24 stress-controlled measurements.

For the strain-controlled creep–fatigue interaction (ECFI) tests, the triangular loading waveform had a strain ratio of *R* = 0, as illustrated in Figure 3b. Maximum strains of 1.0%, 1.2%, 1.4%, and 2.0% were selected with reference to the maximum stresses used in the SCFI tests. Dwell times of 0 and 30 s were applied at the maximum strain; the 0 s condition corresponds to strain-controlled pure fatigue (EPF) testing. The original experimental design used one specimen for each strain-controlled condition to provide a preliminary comparison across four maximum strain levels, whereas the stress-controlled series was replicated to characterize its anticipated scatter. Consequently, the strain-controlled results are descriptive and do not support statistical significance claims. Table 4 summarizes the eight strain-controlled tests.

## 3. Experimental Results and Discussion

### 3.1. Stress–Strain Response

Figure 4 shows representative experimental stress–strain hysteresis loops for FGH95 under stress- and strain-controlled loading. Figure 4a presents loops from stress-controlled tests at *σ*_max_ = 1260 MPa with dwell times of 0 and 30 s. Under both conditions, the maximum strain increases with cycle number, indicating progressive ratcheting deformation and the accumulation of inelastic strain. During the early cycles, the loops translate toward higher strain as the repeated asymmetric loading produces a nonzero strain increment in each cycle. The translation continues through the middle stage and becomes more rapid near failure. The 30 s dwell increases the strain accumulated during each hold, makes the ratcheting response more pronounced, and produces a larger final strain than the no-dwell condition. Figure 4b presents loops from strain-controlled tests at *ε*_max_ = 1.4% with dwell times of 0 and 30 s. Unlike the stress-controlled case, for which the minimum stress remains 0 MPa, strain-controlled loading at *R* = 0 produces compressive stress when the strain returns to zero. The compressive branch is therefore an inherent part of the imposed zero-to-maximum-strain cycle. The 30 s tensile dwell causes stress relaxation at the maximum strain and increases the magnitude of the subsequent compressive stress. Consequently, the loop becomes wider and its enclosed area increases relative to that in the no-dwell test, indicating greater energy dissipation per cycle. Its influence on fatigue life, however, depends on the imposed maximum strain, as discussed in Section 3.3.1.

To further examine the stress evolution of FGH95 alloy with the number of cycles under EPF and ECFI tests, the stress amplitude *σ*_a_ and mean stress *σ*_m_ versus cycle number N were extracted at four maximum strains (1.0%, 1.2%, 1.4%, and 2.0%), as shown in Figure 5. From Figure 5a, it can be seen that as the maximum strain *ε*_max_ increases from 1.0% to 2.0%, the stress amplitude *σ*_a_ increases from about 870 MPa to approximately 1200 MPa. Under the four maximum strains, the evolution of the stress amplitude with the number of cycles shows different trends. For maximum strains of 1.0% and 1.2%, the stress amplitude exhibits slight softening in the early stage (about first 10 cycles), followed by gradual hardening, and then gradual softening until failure after more than 100 cycles. For maximum strains of 1.4% and 2.0%, the FGH95 alloy exhibits initial hardening followed by gradual softening until failure. From the mean stress evolution shown in Figure 5b, the mean stress decreases gradually with increasing maximum strain, which is a result of the Bauschinger effect. According to the study by Qin et al. [6], for FGH95 under different maximum strains, the rate of mean stress decrease in the early life (about first 10% of the fatigue life) is essentially the same. The subsequent stress relaxation is related to the maximum strain: under the small maximum strain of 1.0%, the mean stress continues to relax, whereas under maximum strains of 1.2%, 1.4%, and 2.0%, the mean stress relaxation becomes saturated and does not decrease further as the number of cycles increases.

From Figure 5c, which shows the evolution of stress amplitude with a 30 s dwell, it can be seen that under dwell conditions the stress amplitude increases from about 960 MPa at a maximum strain of 1.0% to approximately 1350 MPa at 2.0%, both of which are higher than the corresponding values of about 870 MPa and 1200 MPa in the no-dwell case. This indicates that dwell at the maximum strain increases the stress amplitude and promotes damage evolution, which is consistent with the larger hysteresis loop area observed under dwell conditions in Figure 4b. The cyclic hardening/softening behavior is generally similar with and without dwell: with increasing maximum strain, the initial cyclic softening becomes weaker while the cyclic hardening becomes stronger, and in the later stage, all cases exhibit cyclic softening. In Figure 5d, the initial mean stresses under dwell conditions for maximum strains from 1.0% to 2.0% are 153.8 MPa, 109.5 MPa, 63.2 MPa, and 12.9 MPa, respectively, which are much smaller than the corresponding values of 300.3 MPa, 237.1 MPa, 176.6 MPa, and 37.3 MPa in the no-dwell case. Stress relaxation at the maximum strain increases the compressive stress during subsequent unloading and therefore reduces the mean stress. Compared with the no-dwell cases, the mean stress relaxation under dwell loading is more consistent among the four maximum strain levels over the fatigue life, although the mean stress fluctuations are larger. A plausible interpretation, consistent with published studies of nickel-based superalloys, is that thermally activated recovery and precipitate evolution compete with cyclic plasticity-induced hardening and thereby contribute to the observed stress fluctuations [26]. 

Figure 6 presents the SCFI results together with the PC results. Figure 6a shows strain versus cycle number for SCFI tests with a 30 s dwell. On a log–log scale, the strain increases slowly at a nearly stable rate during the early and middle stages and then accelerates progressively until complete fracture. This three-stage form becomes clearer when the accumulated strain is plotted over the entire life. Figure 6b plots the SCFI strain against accumulated dwell time and superimposes these data on the PC curves at the same maximum stresses. Both types of tests show a relatively steady early-stage increase followed by rapid late-stage growth, which permits direct comparison of deformation accumulated during interrupted and continuous loading. Figure 6c enlarges the initial loading period. The PC tests show a more pronounced two-stage response, with a rapid initial strain increase followed by a relatively stable increase, whereas the SCFI tests show a smaller initial strain increment. The difference indicates that creep accumulated during repeated 30 s holds is not identical to that accumulated during uninterrupted PC loading, even when the nominal maximum stress is the same. One literature-consistent interpretation is that cyclic loading increases dislocation interactions and back stress, thereby reducing the effective driving stress for creep during the dwell, while repeated unloading interrupts continuous creep deformation [12]. As cycling continues, the strain growth rate remains lower than the corresponding PC rate for much of the life. At the final stage, however, SCFI strain increases rapidly and ultimately exceeds the PC strain. This behavior may reflect the combined effects of cyclic softening, recovery of the deformation substructure, and damage-induced reduction of the effective load-bearing area [3], together with continuous ratcheting strain accumulation. The asymmetric stress waveform maintains a positive ratcheting increment, and the accumulated deformation increases the local strain before final fracture.

### 3.2. Creep Strain Evolution

During the dwell stage, ratcheting under stress control and stress relaxation under strain control are clearly visible. The strain increment during each dwell was defined as the creep strain *ε*_c_, as illustrated in Figure 3. For the SCFI tests, *ε*_c_ in each cycle was measured directly from the hysteresis loop (Figure 3a); for the ECFI tests, *ε*_c_ was calculated using the following equation:

(1) $$\varepsilon_{c}=\frac{\sigma_{\max}-\sigma_{\mathrm{ed}}}{E}$$

where *σ*_max_, *σ*_ed_ denote the stresses at the beginning and end of the dwell time, respectively, and *E* denotes the Young’s modulus of this material at the test temperature. The creep strain evolutions in all SCFI and ECFI tests were then obtained and are presented in Figure 7. In the SCFI test shown in Figure 7a, the creep strain during the first hold cycle is the largest; it then decreases rapidly with increasing cycle number, and upon further cycling, the decreasing trend decelerates and eventually reverses to an increase. This evolution appears to contradict the cyclic hardening behavior presented in Figure 5. The ECFI test results in Figure 7b exhibit essentially the same characteristics: the creep strain first decreases and gradually stabilizes and then remains nearly constant with a slight increase in the mid-to-late stage of life.

The decrease in creep strain during the initial hold periods, followed by a subsequent increase, is compatible with the overall cyclic hardening–softening response of FGH95. A plausible interpretation, consistent with the internal-stress evolution reported for a nickel-based alloy [27], is that early cyclic deformation increases dislocation interactions and back stress, which reduces the effective stress driving creep and decreases the hold-period creep strain. With continued cycling, thermally activated recovery and interactions between dislocations and γ′ precipitates may weaken resistance to deformation [28], while back stress approaches saturation or relaxes. Cavity or microcrack development may also accelerate inelastic straining late in life [2].

According to the tensile curve, stresses of approximately 1200 and 1260 MPa correspond to strains of approximately 1.0% and 2.0%, respectively. The creep strains under these two stress-controlled and two strain-controlled conditions are compared in Figure 8. Figure 8a presents the creep strain at different cycle numbers, and Figure 8b shows the cumulative creep strain. In the early stage, the creep strains are comparable between SCFI at *σ*_max_ = 1200 MPa and ECFI at *ε*_max_ = 1.0%. As cycling continues, the SCFI creep strain becomes larger than the ECFI value. The early-stage creep strains are also broadly comparable between SCFI at *σ*_max_ = 1260 MPa and ECFI at *ε*_max_ = 2.0%; however, with continued cycling, the ECFI creep strain becomes considerably larger than the SCFI value. Thus, the relative magnitude reverses with loading level: SCFI produces the larger creep strain at the lower equivalent level, whereas ECFI produces the larger creep strain at the higher equivalent level. The cumulative creep strain in Figure 8b shows the same load-dependent reversal. Thus, neither control mode produces uniformly greater creep strain across the investigated range. The association between increasing creep strain and decreasing life indicates an increasing contribution of time-dependent deformation.

### 3.3. Life in Different Creep–Fatigue Tests

#### 3.3.1. Fatigue Life Behavior

All test results are summarized in Table 3 and Table 4, together with the accumulated dwell time under each loading condition. Under stress control with a 30 s dwell, the mean fatigue life decreases from 137,031, 51,824, 15,389, and 1979 cycles without dwell to 2221, 1029, 316, and 73 cycles, respectively, corresponding to reductions of 98.4%, 98.0%, 97.9%, and 96.3%. The accumulated dwell times in the SCFI tests are 1.59, 1.71, 1.01, and 1.16 times the corresponding mean PC rupture times. Figure 9 presents maximum stress versus cycle number (S–N) and maximum stress versus accumulated dwell time (S–T) on log–log axes, together with fitted curves. The differences between the SCFI results and the SPF and PC results indicate that the 30 s dwell strongly changes the damage accumulation under stress control. The mean PC rupture time is especially sensitive to stress at 1200 and 1260 MPa, close to the measured yield strength of FGH95 (1169 MPa). The stress-controlled values reported as averages are arithmetic means of two specimens. The duplicate data show appreciable variability, particularly for SPF at 1340 MPa (4323–26,455 cycles) and SCFI at 1260 MPa (598–1460 cycles). With only two stress-controlled replicates per condition and one strain-controlled specimen per condition, population variability and statistical significance cannot be estimated formally; the following comparisons are therefore interpreted as descriptive trends, which is a limitation of the current dataset.

Table 4 shows that, as the maximum strain increased from 1.0% to 2.0%, the measured EPF lives were 3343, 1650, 849, and 189 cycles, whereas the corresponding ECFI lives with a 30 s dwell were 3632, 1047, 509, and 69 cycles. The observed life changes were −8.6%, 36.5%, 40.0%, and 63.5%, where the negative value denotes the measured increase at 1.0%. Two observations follow from these data. First, the response depends on the imposed maximum strain: the dwell reduces the measured life at 1.2–2.0%, with a larger reduction at higher maximum strain, whereas the 1.0% measurements change from 3343 to 3632 cycles. Because each strain-controlled condition was represented by one specimen, this difference is reported descriptively and is not interpreted as statistically significant. Second, the observed dwell-induced life reductions in the ECFI tests at 1.2–2.0% are less severe than the corresponding reductions in the SCFI tests.

For the EPF and ECFI tests, Figure 10a plots maximum strain against life on log–log axes. At maximum strains of 1.2%, 1.4%, and 2.0%, the measured life is lower with the 30 s dwell, whereas at 1.0% it changes from 3343 to 3632 cycles. Figure 10b plots maximum strain against total dwell time. For comparison, the mean total dwell times for SCFI and PC at 1200 and 1260 MPa, corresponding approximately to maximum strains of 1.0% and 2.0%, are also shown. Maximum strain and total dwell time exhibit an approximately linear relationship on log–log axes. At the lower equivalent level (1.0% and 1200 MPa), ECFI produces a greater total dwell time than SCFI; at the higher equivalent level (2.0% and 1260 MPa), the opposite ordering is observed.

#### 3.3.2. Creep–Fatigue Interaction

During the dwell, ECFI tests exhibit stress relaxation, whereas SCFI tests exhibit creep strain accumulation. Characterizing these responses helps evaluate the time-dependent contribution to creep–fatigue damage and provides a basis for life modeling. Figure 11a shows in detail the stress relaxation response during the maximum strain dwell at the half-life cycle. At all four maximum strain levels, the stress decreases rapidly at the beginning of the dwell and then approaches a lower relaxation rate. For maximum strains up to 1.4%, relaxation approaches saturation before 30 s; at 2.0%, the stress continues to relax throughout the 30 s dwell. The relaxation magnitudes at maximum strains of 1.0%, 1.2%, 1.4%, and 2.0% are 9.4, 20.7, 41.7, and 161.3 MPa, respectively. Thus, relaxation is limited at maximum strains below 1.4% but becomes much more pronounced at 2.0%. The stress history during the dwell also shows why a constant-stress creep reference cannot directly represent the entire ECFI hold. Figure 11b shows creep elongation during the maximum stress dwell in the SCFI tests. In contrast to the ECFI stress response, the creep elongation increases continuously throughout the dwell. Both the creep-elongation rate and the total elongation increase monotonically with maximum stress, and no evident saturation tendency is observed within 30 s at any of the four stress levels. Consequently, stress-controlled loading maintains the maximum stress during the dwell while strain-controlled loading progressively relaxes the stress. This difference is consistent with the larger observed life reduction in the SCFI series, although the limited number of specimens precludes a statistical comparison between control modes.

In creep–fatigue interaction tests, the relative contributions of creep and fatigue are often approximated using the time fraction rule (TFR), also termed linear damage summation (LDS). The nominal creep fraction is the ratio of accumulated dwell time in a coupled test to rupture time in a PC test, and the nominal fatigue fraction is the ratio of cycles in the coupled test to cycles in a PF test. The nominal total fraction is the sum of these two terms. Table 5 summarizes these calculated fractions. For the strain-controlled cases, the PC data at 1200 and 1260 MPa were used as reference conditions for ECFI at maximum strains of 1.0% and 2.0%, respectively; no nominal creep fraction was calculated at 1.2% or 1.4%. These fractions are outputs of the TFR/LDS approximation and are not direct measurements of physical creep or fatigue damage.

For the SCFI tests, Table 5 gives nominal creep fractions of 1.59, 1.71, 1.02, and 1.15 at 1200, 1260, 1340, and 1430 MPa, respectively, whereas the nominal fatigue fractions range from 0.02 to 0.04. The corresponding nominal totals are 1.61, 1.73, 1.04, and 1.19 and therefore exceed unity at every stress level. Within the TFR/LDS framework, these values show that the accumulated dwell time is comparable with or greater than the reference PC rupture time, while the number of cycles is only a small fraction of the SPF life. The relatively larger totals at 1200 and 1260 MPa result from the long-accumulated dwell times compared with the mean PC rupture times at those stresses. At 1340 and 1430 MPa, both the CFI life and accumulated dwell time are much shorter, but the same qualitative partition is obtained. These calculated fractions are useful for demonstrating how the conventional rule treats the experimental histories. They should not, however, be interpreted as direct measurements showing that the coupled loading physically suppresses one damage mode or improves creep damage resistance. The early SCFI strain accumulation in Figure 6b is lower than that in PC, whereas the late-stage strain increases rapidly.

For the ECFI tests, the nominal creep fraction at a maximum strain of 1.0% is 2.61 and the nominal fatigue fraction is 1.09. These numerical values do not demonstrate that physical creep damage predominates; instead, the creep fraction is inflated because the method counts the entire 30 s dwell as creep exposure at a fixed reference condition even after the stress has largely relaxed. The value greater than unity and the nominal total of 3.69 demonstrate the inapplicability of this approximation to the present strain-controlled condition. At a maximum strain of 2.0%, the nominal creep and fatigue fractions are 1.10 and 0.37, respectively, but the same qualification applies. The physical interpretation should therefore rely on the measured stress relaxation and creep strain responses, while Table 5 is retained to illustrate the limitation of the conventional method.

For both control modes, the nominal fatigue fraction decreases and the nominal creep fraction increases as the applied stress or maximum strain increases. This calculation is qualitatively consistent with the increase in creep strain shown in Figure 7, but it does not establish the actual partition of physical damage. The discrepancy between the nominal fractions and the measured deformation response motivates use of a strain-based descriptor in the life model developed below.

## 4. Creep–Fatigue Life Prediction

### 4.1. Unified Strain-Based CFI Life Prediction Model

For high-temperature fatigue life prediction, models based on the Manson-Coffin relation, differential approaches, and linear damage-accumulation criteria have been widely studied. These methods relate failure life to stress, strain, energy, time fraction, or combinations of these quantities, but their parameters and assumptions are often tied to a particular loading waveform. Luo et al. [29] used the Basquin model to predict the notch-fatigue life of FGH95 at 420 and 600 °C. Their work focused on fatigue life assessment for a simulated turbine-disk specimen and demonstrated the importance of representing the local loading level. Chen et al. [30] proposed a life model based on the Walker total strain equation that incorporates creep–fatigue interaction. Wei and Yang [31] investigated the strain-controlled creep–fatigue behavior of the PM superalloys FGH96 and GH4169 at 650 °C, examined the effect of dwell time, and developed a uniaxial fatigue life relation that accounts for both dwell time and loading ratio. Cheng et al. [32] developed a cycle-dependent description of creep–fatigue deformation and life for a nickel-based superalloy at elevated temperature. Wang et al. [33] quantified creep–fatigue damage using a cycle-by-cycle strain energy density exhaustion approach and showed that the effects of strain ratio and dwell time on life can be described by an energy-based model. These studies provide important bases for high-temperature life assessment. Most existing life-prediction methods, however, are developed and calibrated for cyclic loading under a single control mode. Their applicability when the loading history changes between stress control and strain control remains uncertain, particularly because ratcheting and stress relaxation generate different time-dependent strain histories. Predicting creep–fatigue life across control modes is therefore more difficult, and a unified formulation for stress- and strain-controlled loading is still needed.

Creep–fatigue life is governed by coupled variables that include temperature, imposed strain, dwell duration, and the material’s creep and fatigue resistance. In engineering assessments, these effects are commonly approximated by linear damage summation (LDS), in which separate creep and fatigue fractions are added to form a total damage index [34]. The method is widely used because it requires relatively few material parameters. Its formulation is:

(2) $$D_{LDS}=D_{c-cfi}+D_{f-cfi}=\frac{N_{f-cfi}\cdot t_{d}}{T_{c}}+\frac{N_{f-cfi}}{N_{f-pf}}=1$$

where *D_LDS_* is the total creep–fatigue damage calculated using the LDS rule, $D_{f-cfi}$ is the total fatigue damage, $D_{c-cfi}$ is the total creep damage, $N_{f-cfi}$ represents the creep–fatigue life, $N_{f-pf}$ is the pure fatigue life, *t_d_* is the dwell time per cycle, and *T_c_* is the pure creep rupture time.

The LDS assumption of independent damage fractions does not explicitly describe interaction between creep and fatigue. Skelton et al. [35] introduced a nonlinear damage summation (NDS) formulation to account for this coupling:

(3) $$D_{NDS}=\frac{D_{c-cfi}}{1-D_{f-cfi}}+\frac{D_{f-cfi}}{1-D_{c-cfi}}=1$$

where *D_NDS_* is the total creep–fatigue damage calculated using the NDS rule.

The NDS formulation represents coupling phenomenologically rather than through explicit internal variables. Figure 7 and Figure 8 show that the creep strain range varies with both life and the hardening–softening response measured during PF loading. The interaction is therefore expressed here using strain quantities obtained from the PF, PC, and CFI tests:

(4) $$D_{cfi}=D_{c-cfi}+D_{f-cfi}=\phi D_{c}+\eta D_{f}=\phi \frac{N_{f-cfi}\cdot t_{d}}{T_{c}}+\eta \frac{N_{f-cfi}}{N_{f-pf}}=1$$

where ϕ quantifies the influence of fatigue loading on creep damage in the CFI test, whereas η quantifies the corresponding influence of creep loading on fatigue damage.

For SCFI, ϕ is obtained from the half-life dwell creep rate in the CFI test ${\dot{\varepsilon }}_{c-cfi}$ relative to the corresponding half-life rate in the PC test ${\dot{\varepsilon }}_{c-pc}$. The definition is:

(5) $$\phi =\alpha {\left(\frac{{\dot{\varepsilon }}_{c-cfi}}{{\dot{\varepsilon }}_{c-pc}}\right)}^{p}$$

where *α* and *p* are the fatigue influence coefficient and fatigue influence exponent, respectively. η is evaluated from the half-life dwell creep strain in the CFI test $\Delta \varepsilon_{c-cfi}$ divided by the corresponding half-life ratcheting strain in the PF test $\Delta \varepsilon_{c-cfi}$. Thus:

(6) $$\eta =\beta {\left(\frac{\Delta \varepsilon_{c-cfi}}{\Delta \varepsilon_{f-pf}}\right)}^{q}$$

where *β* and *q* are the creep influence coefficient and creep influence exponent, respectively.

Under strain control, ϕ is evaluated using the half-life dwell creep rate from the CFI test ${\dot{\varepsilon }}_{c-cfi}$ relative to the half-life rate in the PC test ${\dot{\varepsilon }}_{c-pc}$. Because stress changes continuously during an ECFI dwell, the coupled test rate is compared with a PC rate evaluated at an equivalent stress. Following Zhang et al. [17], the mean dwell stress is used as this equivalent value:

(7) $$\sigma_{d-cfi}=\frac{\sigma_{0}+\sigma_{ed}}{2}$$

where $\sigma_{d-cfi}$ is the equivalent creep stress for ECFI; $\sigma_{0}$ and $\sigma_{ed}$ are the stresses at dwell start and dwell end, respectively. The PC creep rate at a specified stress is then obtained from Norton’s law:

(8) $$\dot{\varepsilon }=A\sigma^{n}$$

where *A* and *n* are material-dependent creep parameters.

For ECFI, the creep influence factor η is defined using the half-life inelastic strain ratio. Its numerator, $\Delta \varepsilon_{f-cfi}^{in}$ is the CFI nonlinear strain range after removal of the creep strain contribution $\Delta \varepsilon_{c}$; the denominator is the PF nonlinear strain range $\Delta \varepsilon_{f-pf}^{in}$. The resulting expression is:

(9) $$\eta =\beta {\left(\frac{\Delta \varepsilon_{f-cif}^{in}}{\Delta \varepsilon_{f-pf}^{in}}\right)}^{q}$$

(10) $$\Delta \varepsilon_{f-cif}^{in}=\Delta \varepsilon_{f-cif}^{t}-\Delta \varepsilon_{f-cif}^{e}-\Delta \varepsilon_{c-cif}$$

(11) $$\Delta \varepsilon_{f-pf}^{in}=\Delta \varepsilon_{f-pf}^{t}-\Delta \varepsilon_{f-pf}^{e}$$

where $\Delta \varepsilon_{f-cif}^{t}$, $\Delta \varepsilon_{f-cif}^{e}$, $\Delta \varepsilon_{f-pf}^{t}$, and $\Delta \varepsilon_{f-pf}^{e}$ are the total and elastic strain ranges for the CFI and PF tests, respectively. The resulting unified strain-based (USB) model is used to calculate CFI life.

### 4.2. Creep–Fatigue Life Assessment

Data from the PC and PF tests were used to determine the creep and fatigue reference relations required by the proposed USB model. The interaction parameters *α*, *β*, *p*, and *q* were then identified simultaneously by nonlinear least-squares regression using all 12 CFI observations under both control modes. The objective function was the sum of squared logarithmic life residuals, Σ[log_10_(*N_pred,i_*/*N_exp,i_*)]^2^, where *N_pred,i_* and *N_exp,i_* denote the predicted and experimentally measured creep–fatigue lives for the ith observation, respectively, with i = 1, 2, …, 12. This logarithmic formulation gives comparable weight to relative errors across the life range. The fitted parameters are summarized in Table 6, and the calculated lives and error factors are listed in Table 7. For comparison, Table 7 and Figure 12 also include the lives calculated using the conventional TFR model. Because the same 12 CFI observations were used for parameter identification and assessment, the following comparison evaluates calibration fit rather than independent predictive performance.

Table 7 summarizes the calculated lives for the 12 CFI observations. The TFR model uses separate reference relations for the stress- and strain-controlled modes. Four TFR results fall outside the factor-of-two scatter band, and eight (67%) fall outside the factor-of-1.5 band. The USB model uses one set of interaction parameters for both modes. All USB results fall within the factor-of-two band, and three (25%) fall outside the factor-of-1.5 band, indicating closer agreement with the calibration data.

Using a factor-of-two scatter band as an engineering measure of agreement, the USB calculation includes all SCFI and ECFI observations, whereas the TFR model has a maximum error factor of 3.69. For the SCFI data, both methods use the sustained maximum stress during the dwell, although the USB formulation additionally describes the interaction through strain-based quantities. For the ECFI data, however, the maximum strain constraint produces a continuously changing stress history. The largest TFR deviations therefore occur for the ECFI cases because a constant-reference-stress time fraction does not represent progressive stress relaxation during the maximum strain dwell. Only at 2.0%, where relaxation remains active throughout much of the dwell and the equivalent creep contribution is larger, is the TFR error smaller. Figure 12 also shows that the TFR model generally overestimates the observed lives in the present dataset. The difference between the two calculations is thus most evident when the imposed control mode strongly changes the stress during the hold.

Compared with the TFR formulation, the USB model introduces interaction terms based on creep strain rate and inelastic strain. These terms allow the stress- and strain-controlled observations to be represented using a single parameter set rather than two separate control mode-specific damage calculations. Agreement is slightly closer for SCFI than for ECFI, plausibly because the SCFI creep rate can be obtained directly from strain accumulation during the maximum stress dwell. ECFI, by contrast, requires an equivalent-stress approximation to estimate creep strain rate from the stress relaxation history. This approximation introduces additional uncertainty between the calculated equivalent rate and the actual time-dependent response. The remaining deviations in Table 7 are therefore not identical for the two modes, even though all USB results lie within the factor-of-two band. Within the calibration dataset, the USB model provides closer agreement than the TFR model across both control modes. This result supports the use of strain-based interaction descriptors for correlating the present dataset.

## 5. Conclusions

In this study, strain-controlled creep–fatigue tests with maximum strains of 1.0–2.0% and stress-controlled tests with maximum stresses of 1200–1430 MPa were conducted on the PM nickel-based superalloy FGH95 at 620 °C, with and without a 30 s tensile dwell. The alloy is used in aero-engine turbine disks and can experience cyclic loading together with high-temperature load holds. The cyclic stress–strain response, ratcheting deformation, stress relaxation, creep strain evolution, accumulated dwell time, and fatigue life behavior under the two control modes were evaluated. A unified strain-based life model was calibrated for the available CFI dataset and compared with the conventional time fraction approach. The principal conclusions are as follows:

Regardless of control mode, pure fatigue loading produces cyclic hardening, subsequent softening, and final fracture. Under ECFI loading, the tensile hold introduces time-dependent deformation; however, stress relaxation during the hold period increases the magnitude of compressive stress upon unloading, which changes the subsequent compressive response. Under SCFI loading, the strain ratcheting effect is amplified with increasing dwell time, which not only introduces time-dependent deformation but also is associated with a pronounced reduction in measured life.

The creep strain per cycle initially decreases and then increases with cycle number, corresponding to the transition from cyclic hardening to cyclic softening. The comparison between control modes is load-dependent: SCFI produces greater creep strain at the lower equivalent level (1200 MPa versus 1.0%), whereas ECFI produces greater creep strain at the higher equivalent level (1260 MPa versus 2.0%). Thus, neither control mode produces uniformly greater cumulative creep strain over the investigated range.

The fatigue response of FGH95 to tensile dwell depends strongly on the loading control mode and applied strain level. Under stress control, a 30 s tensile dwell reduced the mean SCFI life by 96.3–98.4%. Under strain control, the effect varied markedly with strain level: at 1.0%, the measured life changed from 3343 cycles without dwell to 3632 cycles with dwell, whereas life reductions of 36.5–63.5% were observed at 1.2–2.0%. Damage fraction analysis further shows that rapid stress relaxation under ECFI causes the conventional TFR/LDS method to overestimate creep damage, as reflected by nominal damage fractions greater than unity.

A unified strain-based creep–fatigue life model was formulated using interaction descriptors based on creep strain rate and inelastic strain. For the 12 CFI observations used in calibration, all USB-calculated lives fall within a factor-of-two scatter band, whereas the TFR model gives a maximum error factor of 3.69. Within the calibration dataset, the strain-based interaction variables provide closer agreement across the two control modes than the TFR calculation.

## Figures and Tables

**Figure 1:**
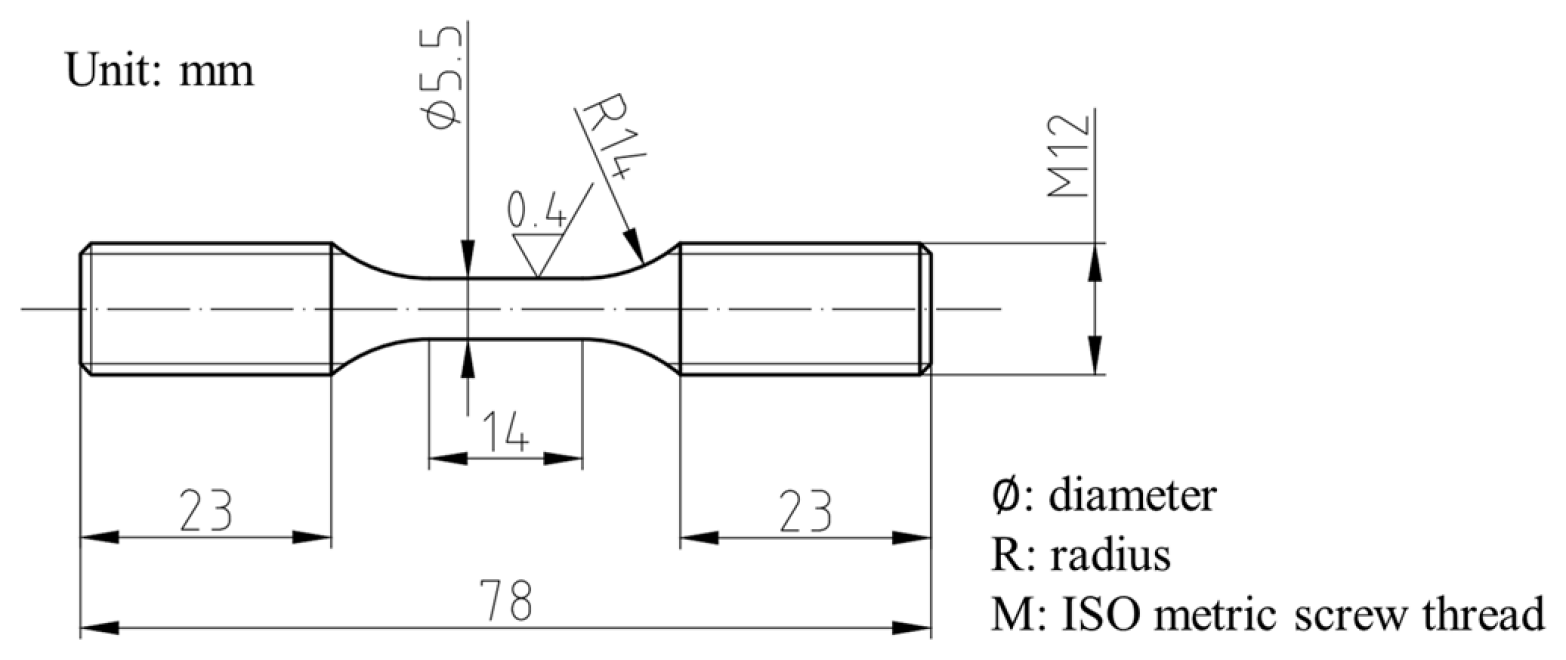
Schematic illustration of test samples.

**Figure 2:**
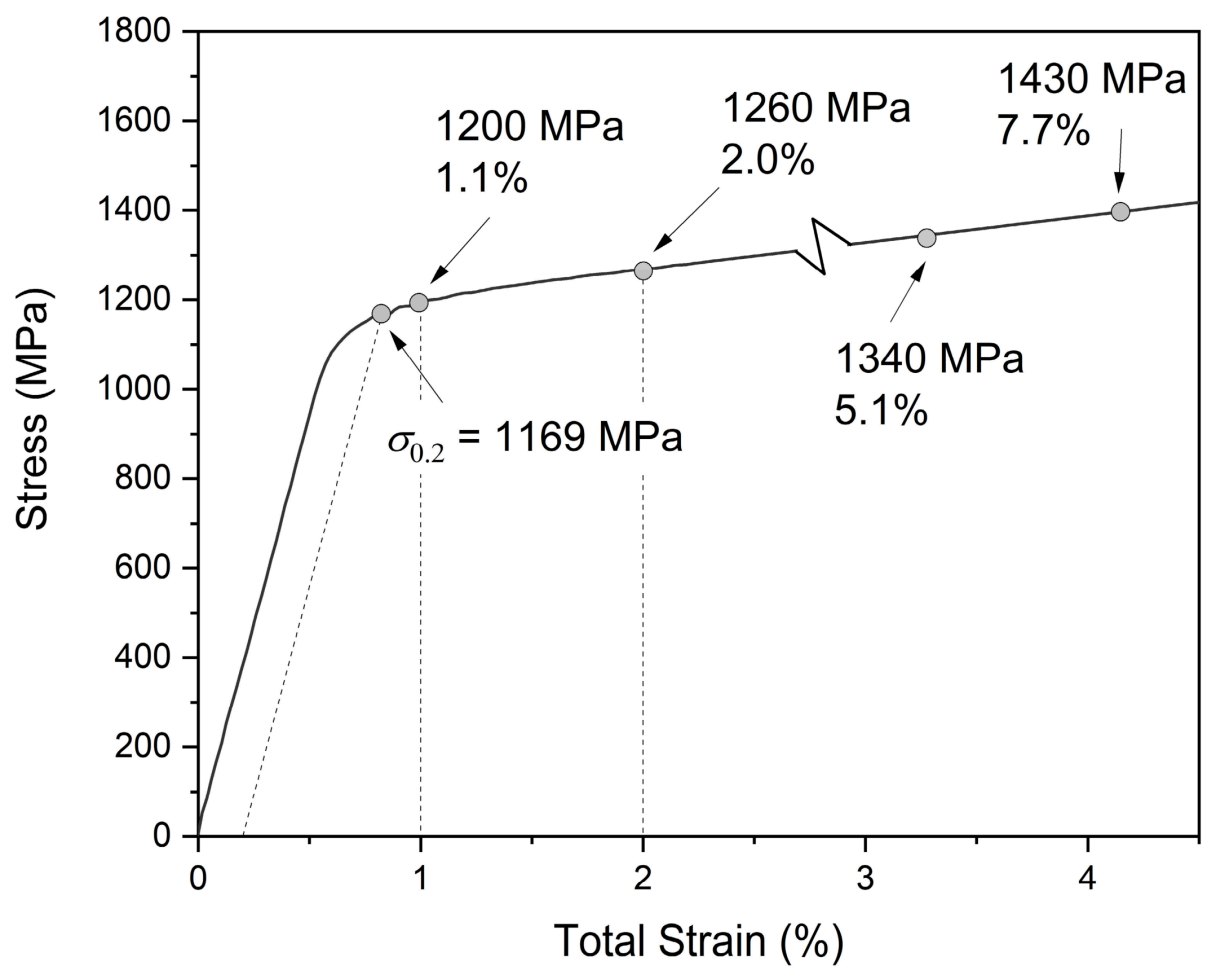
Tensile curve of FGH95 alloy at 620 °C.

**Figure 3:**
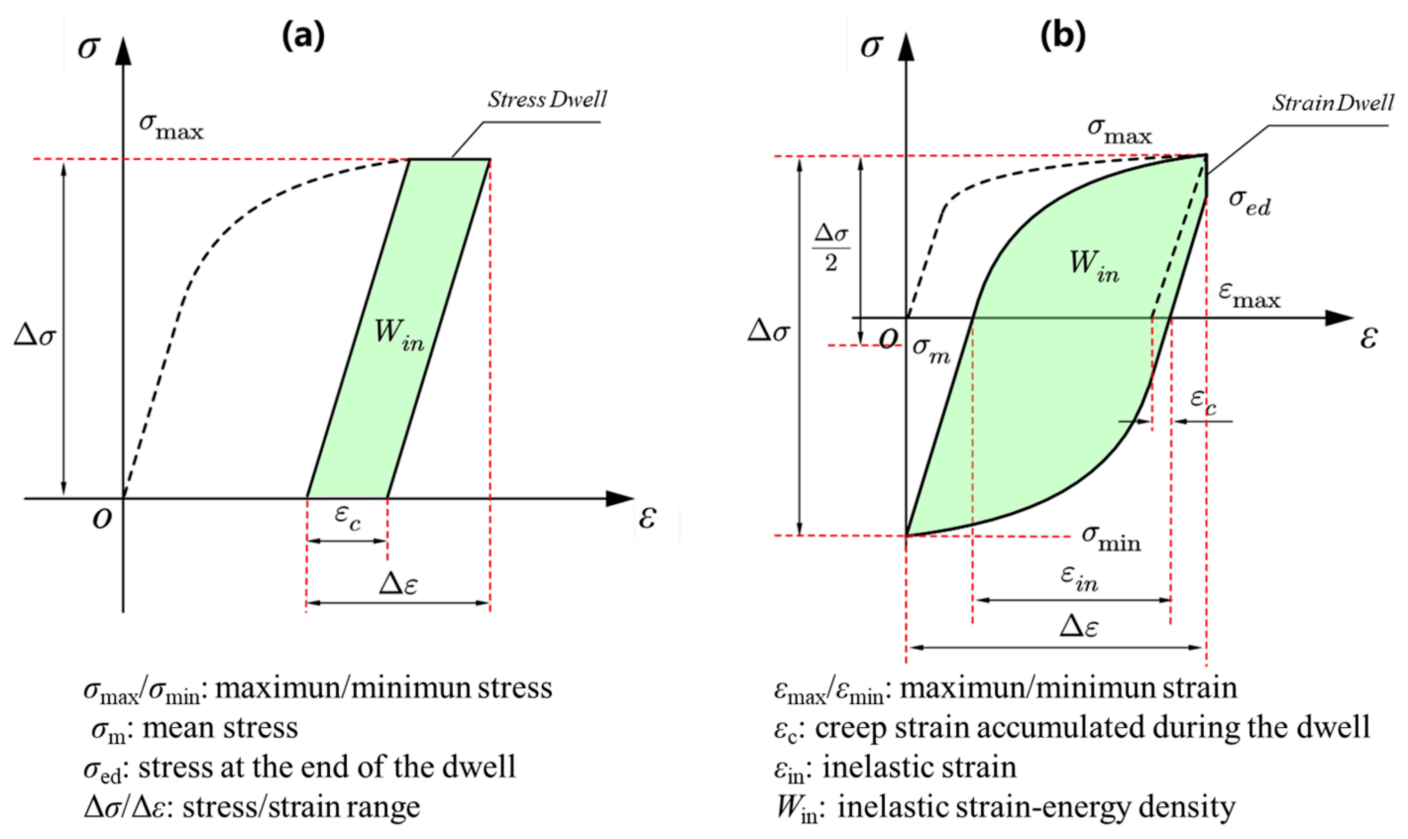
Schematic loading waveforms for CFI tests under (**a**) stress-controlled and (**b**) strain-controlled modes.

**Figure 4:**
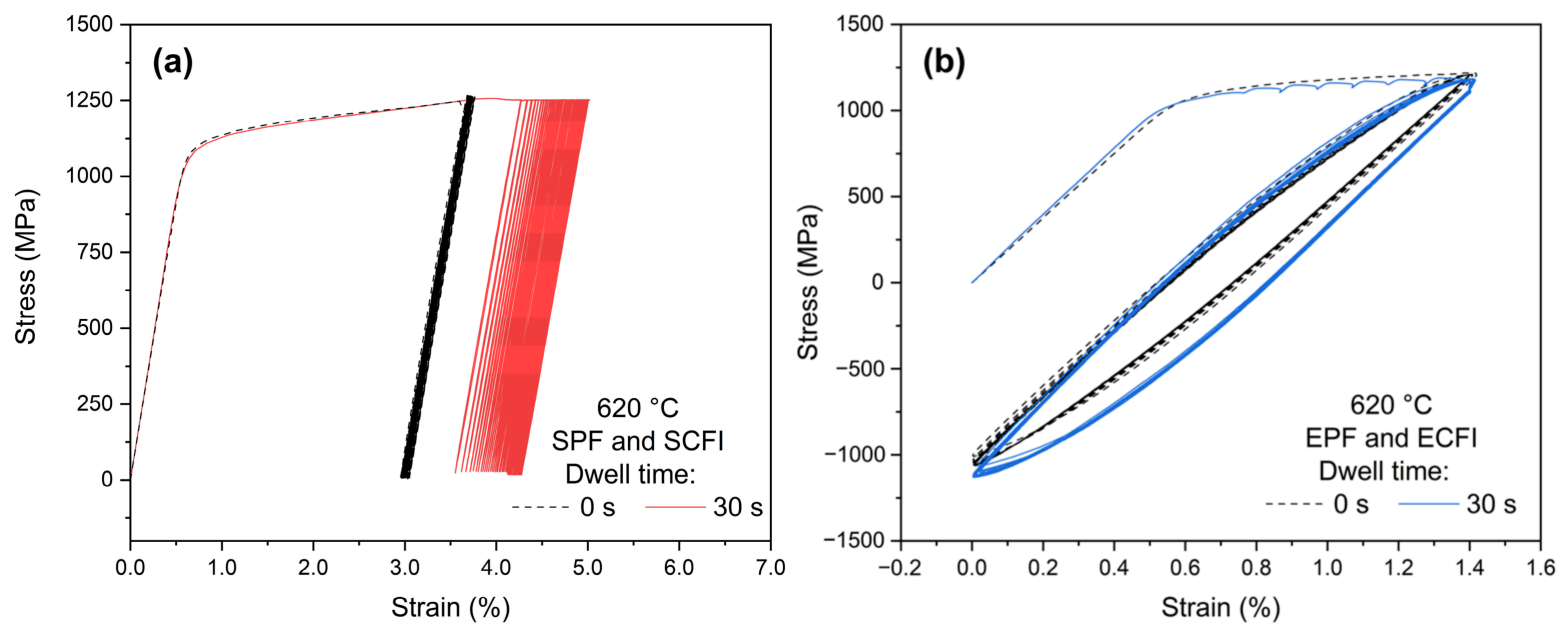
Representative experimental stress–strain hysteresis loops under (**a**) stress-controlled and (**b**) strain-controlled modes.

**Figure 5:**
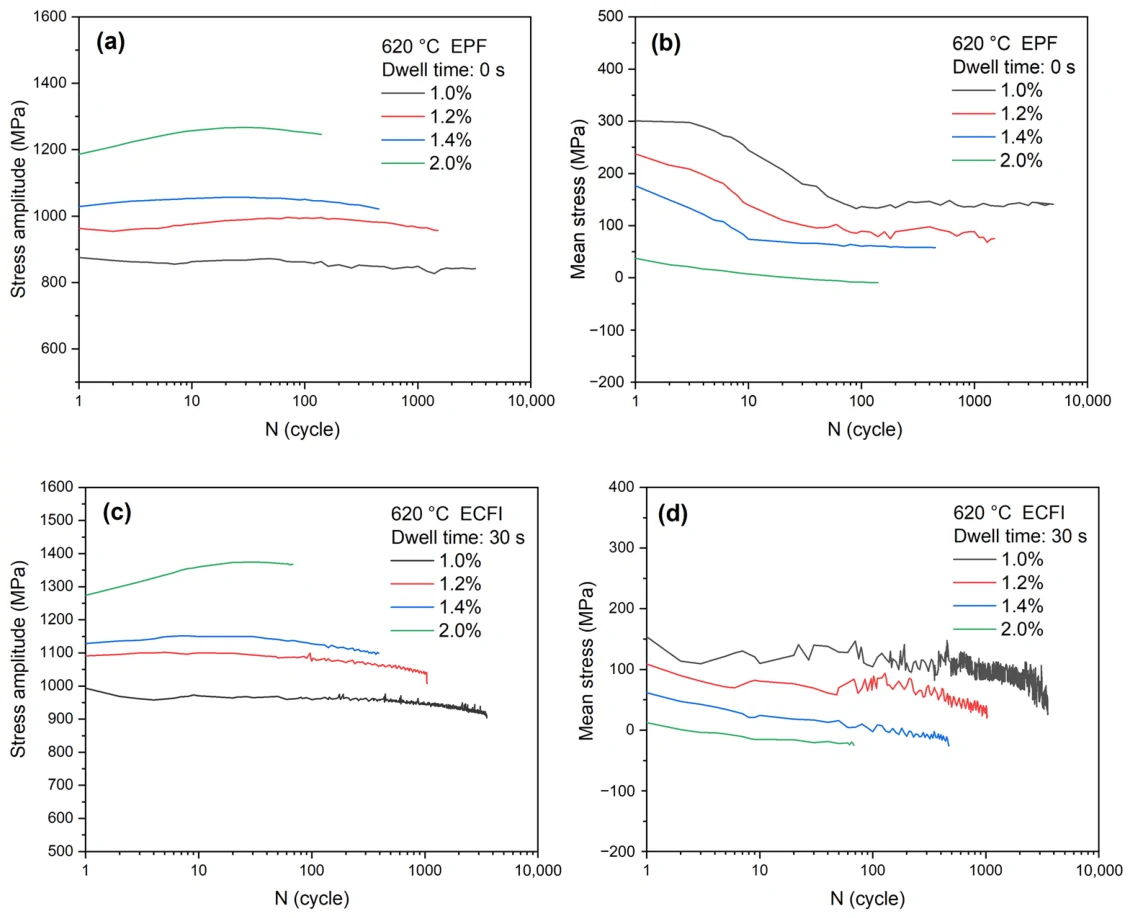
Evolution of stress amplitude and mean stress with cycle number under EPF and ECFI tests: (**a**) stress amplitude *σ*_a_ and (**b**) mean stress *σ*_m_ under EPF; (**c**) stress amplitude *σ*_a_ and (**d**) mean stress *σ*_m_ under ECFI.

**Figure 6:**
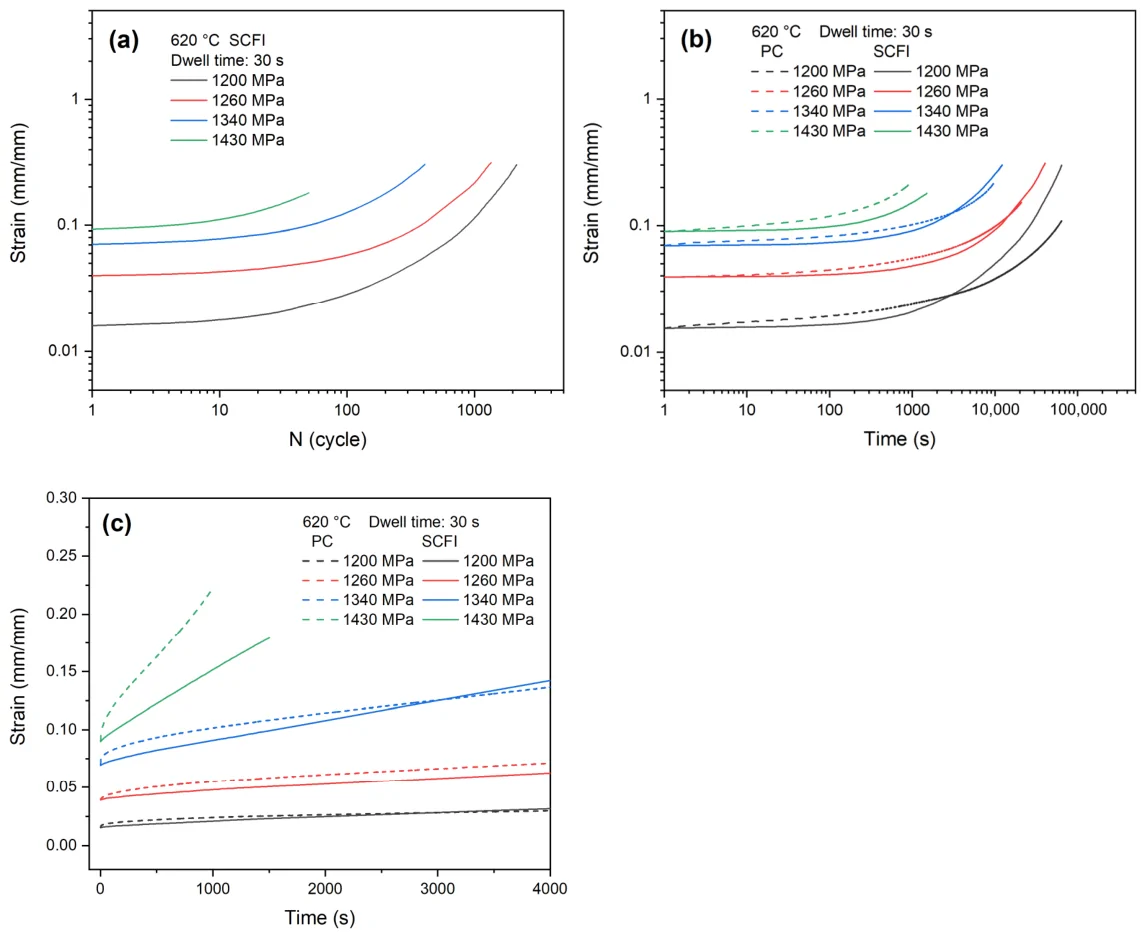
Strain evolution under PC and SCFI tests: (**a**) strain vs. cycles; (**b**) strain vs. time; (**c**) enlarged view of initial stage of strain vs. time curve.

**Figure 7:**
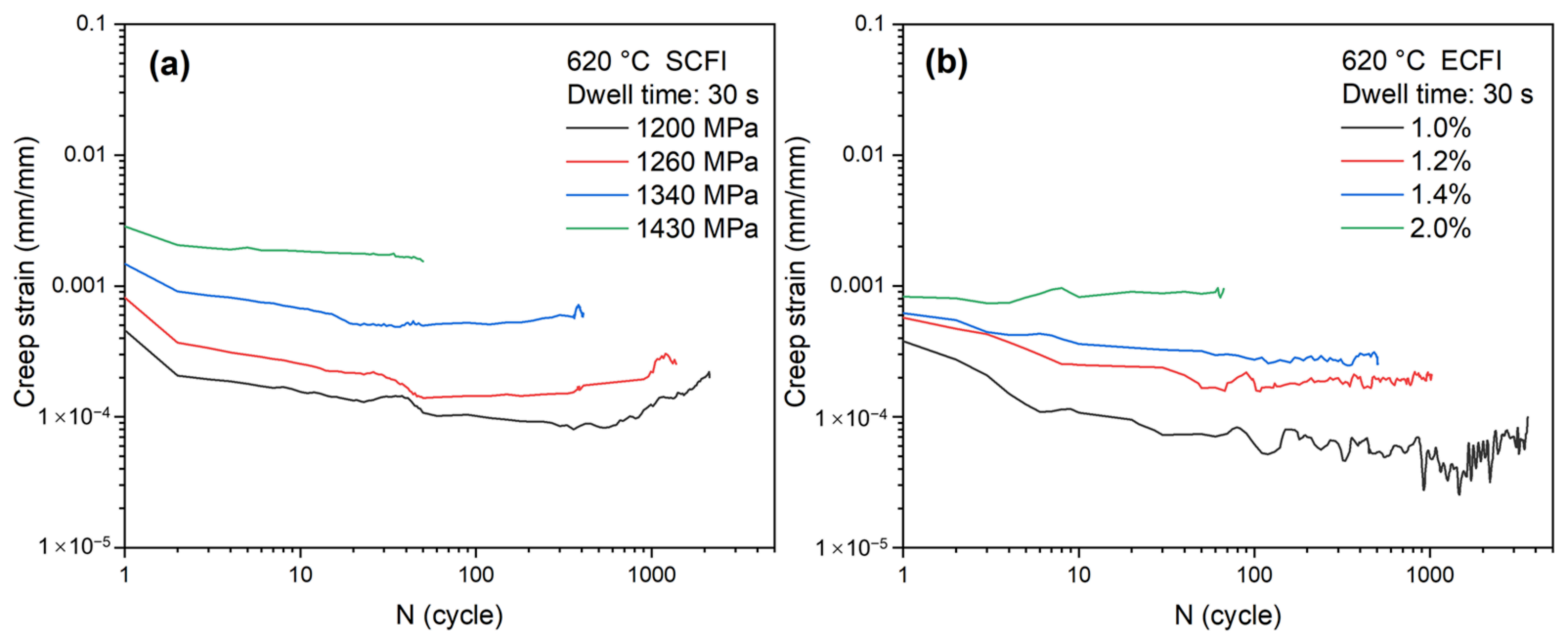
Creep strain evolution under the (**a**) stress-controlled mode and (**b**) strain-controlled mode.

**Figure 8:**
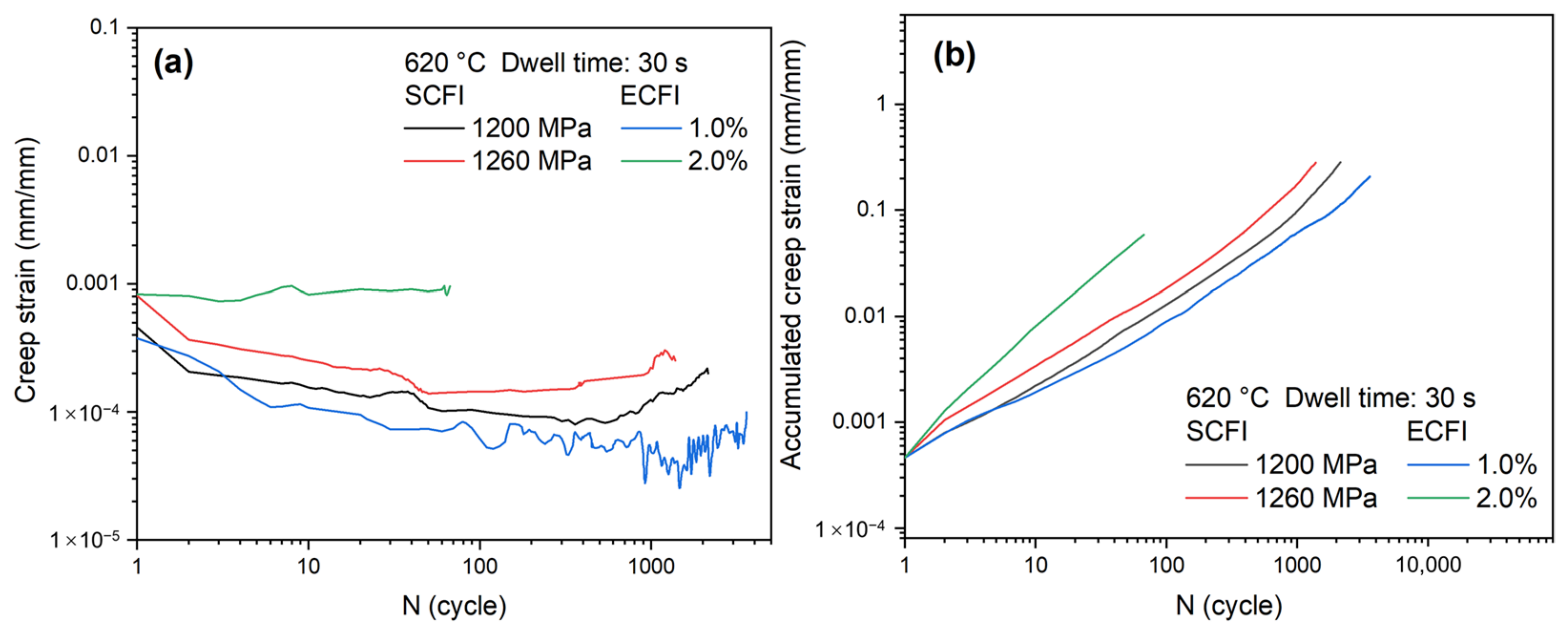
Comparison of creep strain under SCFI and ECFI tests: (**a**) creep strain vs. cycle; (**b**) cumulative creep strain vs. cycle.

**Figure 9:**
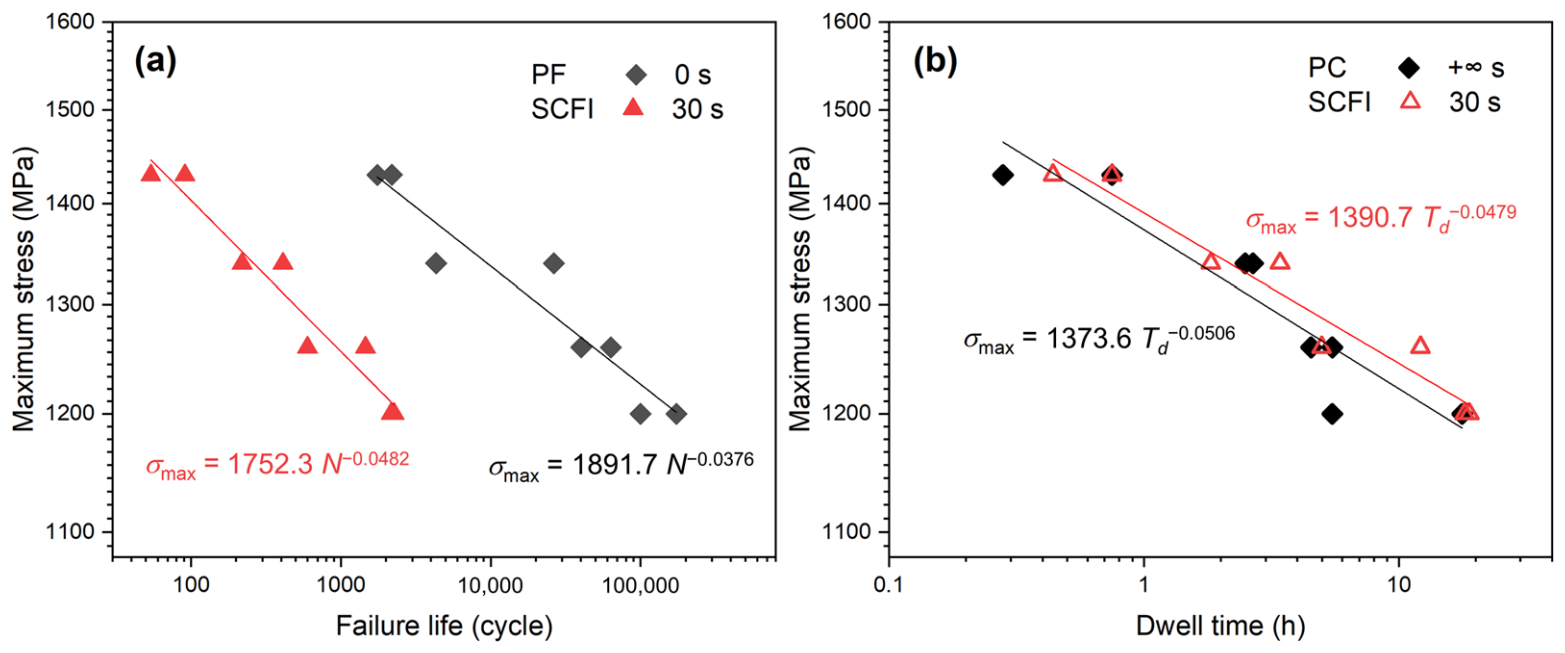
(**a**) Maximum stress versus number of cycles; (**b**) maximum stress versus hold time in the double logarithmic coordinate system.

**Figure 10:**
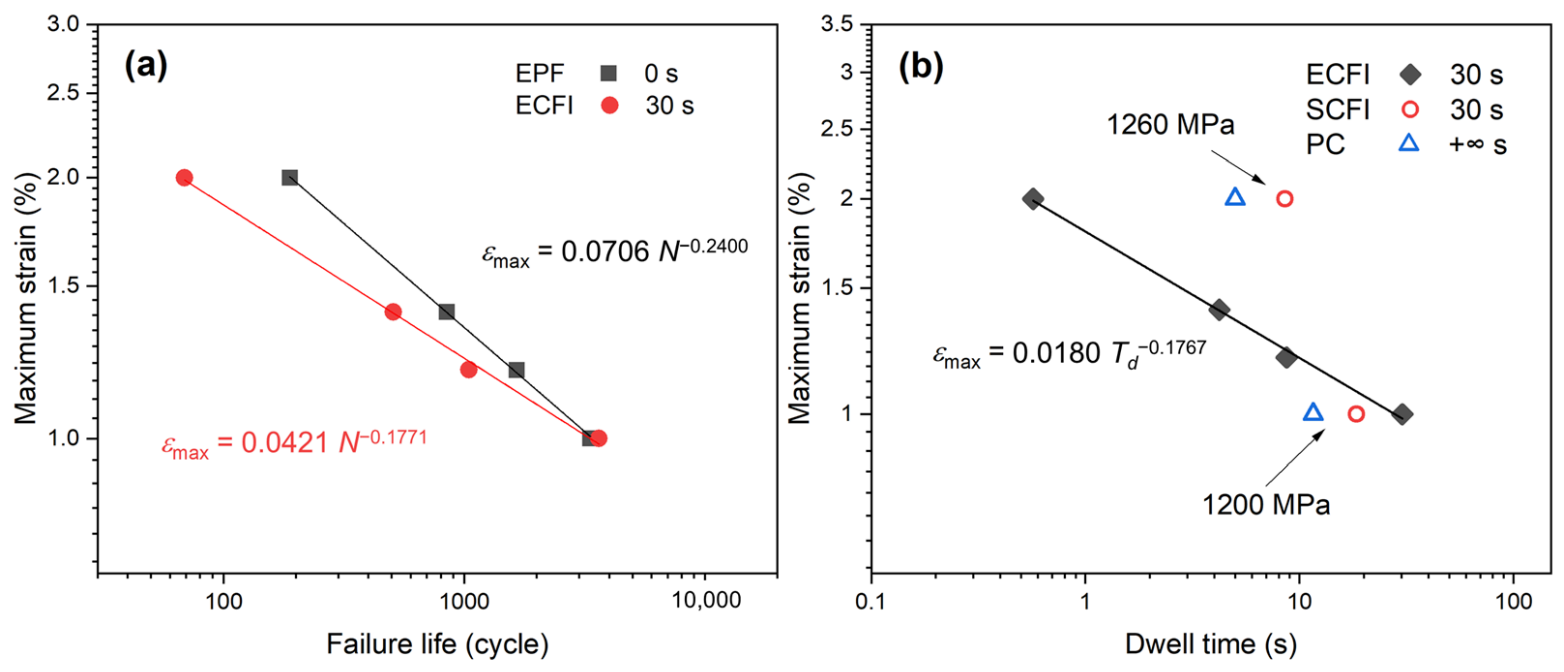
(**a**) Maximum strain versus fatigue life; (**b**) Maximum strain versus dwell time on log–log axes.

**Figure 11:**
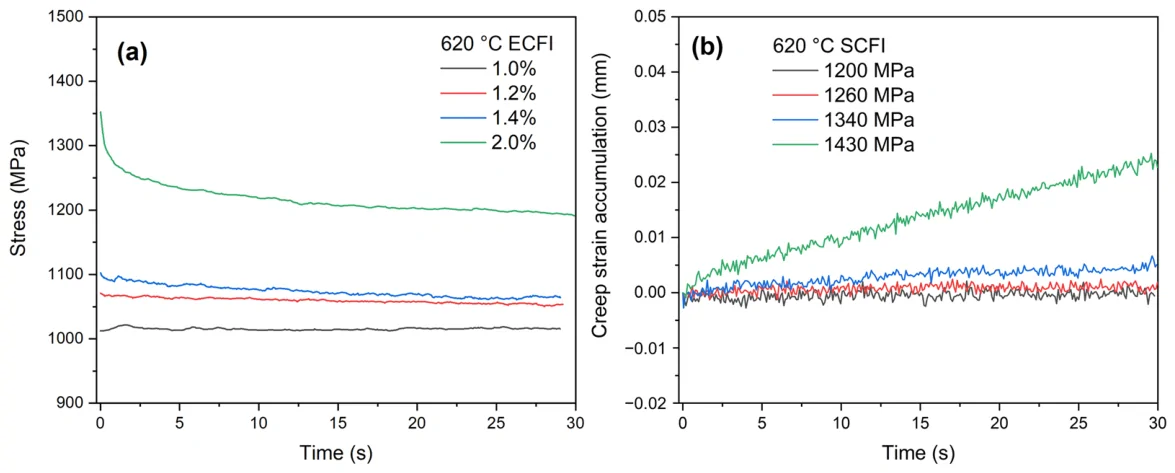
(**a**) Stress relaxation during dwell time in ECFI tests; (**b**) creep strain accumulation during dwell time in SCFI tests.

**Figure 12:**
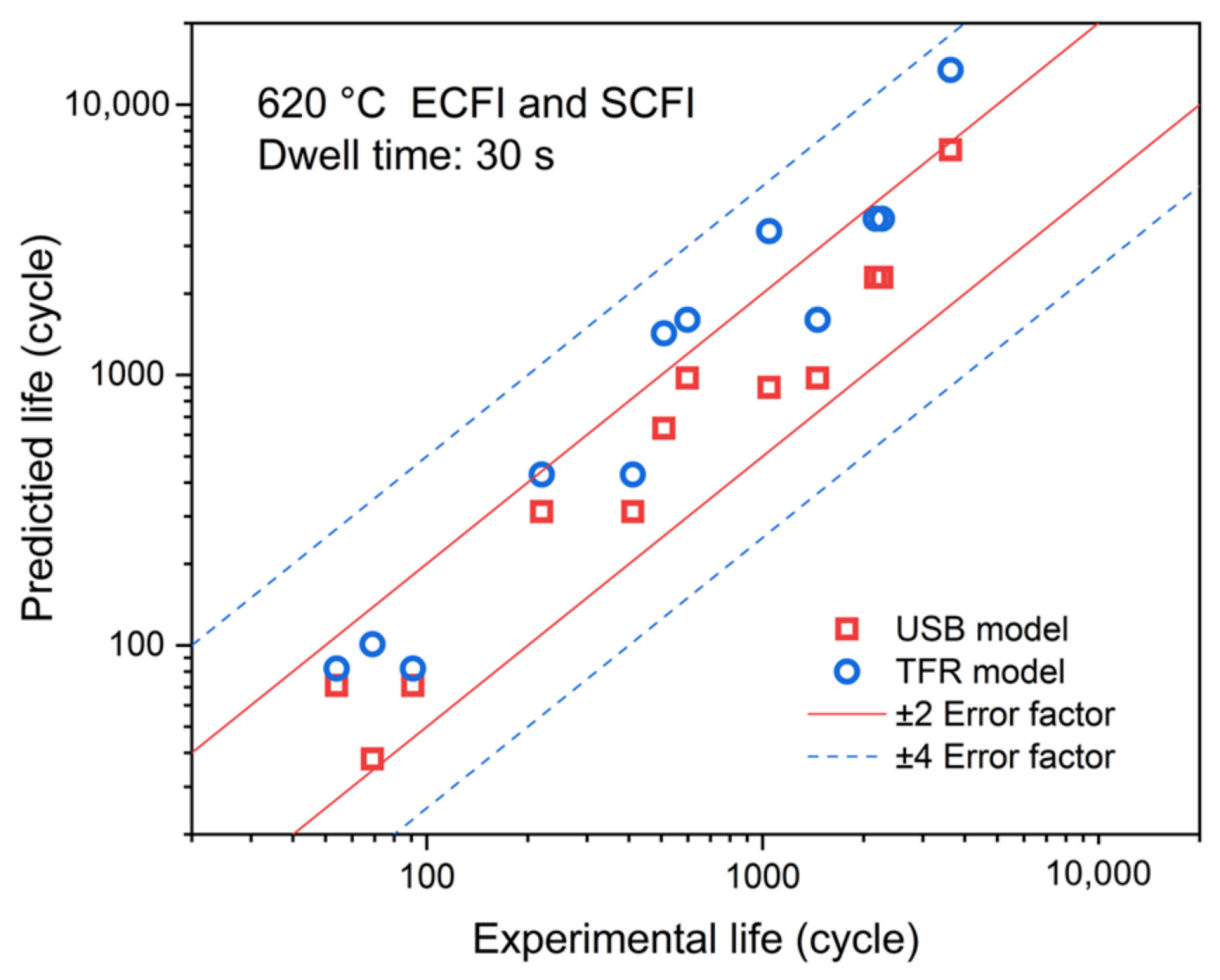
Creep–fatigue life prediction results of FGH95 alloy by different models.

**Table 1:** Chemical compositions (wt.%) of FGH95 superalloy.

Ni	C	Cr	Co	W	Mo	Al	Ti	Nb	B
Bal.	0.04~0.09	12~14	4~9	3.3~3.7	3.3~3.7	3.3~3.7	2.3~2.7	3.3~3.7	0.006~0.015
Zr	Mn	Fe	Ta	Si	P	S	O	N	H
0.03~0.07	0.15	0.5	0.5	0.2	0.2	0.015	0.01	0.005	0.001

**Table 2:** Tensile properties of FGH95 alloy at 620 °C.

Tensile Properties	Value
Elastic modulus/GPa	186.5
Yield strength/MPa	1169
Tensile strength/MPa	1596
Elongation	26.3%
Strain at 1200 MPa	1.1%
Strain at 1260 MPa	2.0%
Strain at 1340 MPa	5.1%
Strain at 1430 MPa	7.7%

**Table 3:** Test parameters under the stress-controlled mode and results of cyclic life.

Test Mode	Maximum Stress/MPa	Holding Time/s	Fatigue Life/Cycle		Accumulated Holding Time/h	
			Sample	Average	Sample	Average
SPF	1200	0	100,002	137,031	0	0
		0	174,060		0	
	1260	0	63,316	51,824	0	0
		0	40,331		0	
	1340	0	26,455	15,389	0	0
		0	4323		0	
	1430	0	1759	1979	0	0
		0	2198		0	
SCFI	1200	30	2173	2221	18.10	18.50
		30	2268		18.89	
	1260	30	598	1029	4.98	8.57
		30	1460		12.16	
	1340	30	411	316	3.42	2.63
		30	220		1.83	
	1430	30	54	73	0.44	0.60
		30	91		0.75	
PC	1200	∞	1	1	5.48	11.61
		∞	1		17.73	
	1260	∞	1	1	5.48	5.01
		∞	1		4.53	
	1340	∞	1	1	2.50	2.59
		∞	1		2.68	
	1430	∞	1	1	0.28	0.52
		∞	1		0.75	

**Table 4:** Test parameters under the strain-controlled mode and results of cyclic life.

Test Mode	Maximum Strain	Holding Time/s	Fatigue Life/Cycle	Accumulated Holding Time/h
EPF	1.00%	0	3343	0
	1.20%	0	1650	0
	1.40%	0	849	0
	2.00%	0	189	0
ECFI	1.00%	30	3632	30.26
	1.20%	30	1047	8.72
	1.40%	30	509	4.23
	2.00%	30	69	0.57

**Table 5:** Damage fractions in creep–fatigue interaction tests.

Stress/Maximum Strain Level	Creep–Fatigue Interaction Tests		Creep Tests	Fatigue Tests	Creep Damage Fraction	Fatigue Damage Fraction	Total Damage
	Total Dwell Time/h	Fatigue Life/Cycle	Rupture Time/h	Fatigue Life/Cycle			
1200 MPa	18.50	2221	11.61	137,031	1.59	0.02	1.61
1260 MPa	8.57	1029	5.01	51,824	1.71	0.02	1.73
1340 MPa	2.63	316	2.59	15,389	1.02	0.02	1.04
1430 MPa	0.60	73	0.52	1979	1.15	0.04	1.19
1.00%	30.26	3632	11.61	3343	2.61	1.09	3.69
1.20%	8.72	1047	/	1650	/	0.64	/
1.40%	4.23	509	/	849	/	0.60	/
2.00%	0.57	69	0.52	189	1.10	0.37	1.46

**Table 6:** Parameters of the proposed model.

**Basic Information**	**Material**	**FGH95**		
	Temperature	620 °C		
Parameters and value	*α*	*β*	*p*	*q*
	0.163	1.633	−0.956	0.850

**Table 7:** Experimental and predicted creep–fatigue lives of FGH95 alloy under different control modes at 620 °C.

Control Mode	Stress/Strain Level	Experimental Life/Cycle	Predicted Life/Cycle		Error Factor	
			TFR Model	USB Model	TFR Model	USB Model
SCFI	1200 MPa	2173	3782	2290	1.74	1.05
		2268			1.67	1.01
	1260 MPa	598	1598	974	2.67	1.63
		1460			1.09	0.67
	1340 MPa	411	428	312	1.04	0.76
		220			1.95	1.42
	1430 MPa	54	82	71	1.52	1.31
		91			0.90	0.78
ECFI	1.00%	3632	13,411	6794	3.69	1.87
	1.20%	1047	3399	899	3.25	0.86
	1.40%	509	1425	634	2.80	1.25
	2.00%	69	101	38	1.46	0.55

## Data Availability

The original contributions presented in this study are included in the article. Further inquiries can be directed to the corresponding author.

## References

[1] Tao Y., Jia J., Liu J. (2010). Microstructure characterization and mechanical properties of FGH95 turbine blade retainers. J. Iron Steel Res. Int..

[2] Tian S., Liu Y., Zhou X., Zhao Z., Bao X., Wang W. (2009). Creep behaviors of FGH95 powder Ni-base superalloy. Chin. J. Aeronaut..

[3] Tian S., Xie J., Zhou X., Qian B., Lun J., Yu L., Wang W. (2011). Microstructure and creep behavior of FGH95 nickel-base superalloy. Mater. Sci. Eng. A.

[4] Xie J., Tian S., Zhou X., Yu X., Wang W. (2012). Influence of heat treatment regimes on microstructure and creep properties of FGH95 nickel base superalloy. Mater. Sci. Eng. A.

[5] Xie J., Tian S., Zhou X. (2012). Effect of HIP temperature on microstructure and creep property of FGH95 alloy. High Temp. Mater. Process..

[6] Qin M., Chen C., Xuan H., Liu Y., Huang B. (2025). A modified cyclic elastoplastic constitutive model considering the variational dynamic recovery term of back stress for FGH95 under asymmetrical cyclic loading. Int. J. Fatigue.

[7] Bernstein H.L. (1979). An Evaluation of Four Current Models to Predict the Creep-Fatigue Interaction in Rene 95.

[8] Li Y., Liu Y., Wu K., Sun J., Zhang D., Fei Q. (2025). Temperature effects on low cycle fatigue deformation of powder metallurgy superalloy FGH95. Int. J. Fatigue.

[9] Hormozi R., Biglari F., Nikbin K. (2015). Experimental and numerical creep-fatigue study of Type 316 stainless steel failure under high temperature LCF loading condition with different hold time. Eng. Fract. Mech..

[10] Chen G., Zhang Y., Xu D., Lin Y., Chen X. (2016). Low cycle fatigue and creep-fatigue interaction behavior of nickel-base superalloy GH4169 at elevated temperature of 650 °C. Mater. Sci. Eng. A.

[11] Mao J., Xiao Z., Hu D., Guo X., Wang R. (2022). A Concise Binomial Model for Nonlinear Creep-Fatigue Crack Growth Behavior at Elevated Temperatures. Materials.

[12] Sun L., Bao X., Guo S., Wang R., Zhang X., Tu S. (2021). The creep-fatigue behavior of a nickel-based superalloy: Experiments study and cyclic plastic analysis. Int. J. Fatigue.

[13] Kalyan B.V., Pavan A., Rao B.S., Narasaiah N. (2024). Effect of stress-hold and strain-hold during Creep-Fatigue Interaction of alloy 617 M. Mater. Today Commun..

[14] Zhao L., Xu L., Han Y., Jing H. (2020). Analysis on stress-strain behavior and life prediction of P92 steel under creep-fatigue interaction conditions. Fatigue Fract. Mater. Struct..

[15] Zhang T., Wang X., Zhou D., Wang R., Jiang Y., Zhang X., Gong J., Tu S. (2022). A universal constitutive model for hybrid stress-strain controlled creep-fatigue deformation. Int. J. Mech. Sci..

[16] Tahir F., Liu Y. (2017). A new experimental testing method for investigation of creep-dominant creep-fatigue interaction in Alloy 617 at 950 °C. Int. J. Press. Vessel. Pip..

[17] Zhang T., Wang X., Ji Y., Zhang W., Hassan T., Gong J. (2020). P92 steel creep-fatigue interaction responses under hybrid stress-strain controlled loading and a life prediction model. Int. J. Fatigue.

[18] Zhang T., Wang X., Zhang C., Jiang Y., Wen J., Zhang X., Gong J. (2023). Revealing the influences of strain amplitudes on hybrid stress–strain controlled creep-fatigue interaction responses for 9%Cr steel. Eng. Fract. Mech..

[19] Zhang C., Chen S., Wang H., Zhong F., Li L., Liu Q., Zhen C., Wang X., Luo L., Bao S. (2024). Creep-fatigue interactive behavior and damage mechanism of TP321 stainless steel under hybrid-controlled conditions. Mater. Charact..

[20] Kang Z., Wang X., Zhou W., Zhang T., Zhou D., Zhao Y., Wen J., Gong J. (2024). Creep-fatigue deformation characteristics and life prediction model of Inconel 718 superalloy under hybrid stress-strain-controlled mode. Fatigue Fract. Mater. Struct..

[21] Kang Z., Wang X., Wang Z., Zhou D., Zhang T., Wen J., Zhang C., Tang J., Gong J. (2024). Cyclic response and residual life prediction of Inconel 718 superalloy after overloading under hybrid stress-strain controlled creep-fatigue loading. Eng. Fail. Anal..

[22] Yuan X., Yu W., Fu S., Yu D., Chen X. (2016). Effect of mean stress and ratcheting strain on the low cycle fatigue behavior of a wrought 316LN stainless steel. Mater. Sci. Eng. A.

[23] Li Z., Qin H., Xu K., Xie Z., Ji P., Jia M. (2023). Fatigue Behavior of the FGH96 Superalloy under High-Temperature Cyclic Loading. Materials.

[24] (2015). Metallic Materials—Tensile Testing—Part 2: Method of Test at Elevated Temperature.

[25] Cui W., Wan J., Yue Z., Yang Z. (2007). Tensile and low cycle fatigue properties of nickel-base powder metallurgy superalloy FGH95. Rare Met. Mater. Eng..

[26] Antolovich S.D. (2015). Microstructural aspects of fatigue in Ni-base superalloys. Trans. R. Soc. A Math. Phys. Eng. Sci..

[27] Wang N., Yu H., Zhao P., Zhang J., Gong J., Xuan F. (2022). Cyclic deformation response of austenitic Ni-based alloy: Mechanical behavior, internal stress evolution and microstructural feature. Mater. Sci. Eng. A.

[28] Zhang L., Zhao L., Roy A., Silberschmidt V., McColvin G. (2019). Low-cycle fatigue of single crystal nickel-based superalloy-mechanical testing and TEM characterisation. Mater. Sci. Eng. A.

[29] Luo P., Peng S., Yu M., Ai X. (2025). Research on simulated specimen of FGH95 gas turbine disc and fatigue life prediction method. Proceedings of the 7th China Aeronautical Science and Technology Conference.

[30] Chen S., Wei D., Wang J., Wang Y., Jiang X. (2020). A new fatigue life prediction model considering the creep-fatigue interaction effect based on the Walker total strain equation. Chin. J. Aeronaut..

[31] Wei D., Yang X. (2009). Investigation and modeling of low cycle fatigue behaviors of two Ni-based superalloys under dwell conditions. Int. J. Press. Vessel. Pip..

[32] Cheng L., Wang R., Wang J., Zhu S., Zhao P., Miura H., Zhang X., Tu S. (2021). Cycle-dependent creep-fatigue deformation and life predictions in a nickel-based superalloy at elevated temperature. Int. J. Mech. Sci..

[33] Wang R., Wang J., Gong J., Zhang X., Tu S., Zhang C. (2018). Creep-fatigue behaviors and life assessments in two nickel-based superalloys. J. Press. Vessel Technol..

[34] Zhang S., Takahashi Y. (2023). Creep-fatigue life and damage evaluation under various strain waveforms for Ni-based Alloy 740H. Int. J. Fatigue.

[35] Skelton R.P., Gandy D. (2014). Creep-fatigue damage accumulation and interaction diagram based on metallographic interpretation of mechanisms. Mater. High. Temp..

